# Brief progress report from the intersocietal working group on differentiated thyroid cancer

**DOI:** 10.1007/s00259-020-04744-8

**Published:** 2020-03-12

**Authors:** Frederik A. Verburg, Sukhjeet Ahuja, Anca M. Avram, Manuel Bardiès, Victor Bernet, Patrick Bourguet, Dagmar Führer-Sakel, Ciprian Draganescu, Gilbert H. Daniels, Bennett Greenspan, Seza Gulec, Laszlo Hegedüs, Jacqueline Jonklaas, Markus Luster, Wim Oyen, Johannes Smit, R. Michael Tuttle, Slimane Zerdoud, Douglas Van Nostrand

**Affiliations:** 1 Department of Nuclear Medicine, University Hospital Marburg, Baldinger Straße, 35043 Marburg, Germany; 2 Society of Nuclear Medicine, Reston, USA; 3 University of Michigan Medical Center, Ann Arbor, MI USA; 4 Centre de Recherches en Cancérologie de Toulouse and UMR 1037, INSERM, Université Toulouse III Paul Sabatier, Toulouse, France; 5 Mayo Clinic College of Medicine, Rochester, FL USA; 6 University Hospital of Martinique, University of Antilles, Pointe-à-Pitre, France; 7 Department of Endocrinology, University Hospital Essen, Essen, Germany; 8 Thyroid Unit and Cancer Center Massachusetts General Hospital, Boston, MA USA; 9 Harvard Medical School, Boston, MA USA; 10 Herbert Wertheim College of Medicine, Florida International University, Miami, FL USA; 11 Department of Endocrinology, Odense University Hospital, Odense, Denmark; 12 Division of Endocrinology, Georgetown University, Washington, DC USA; 13 European Association of Nuclear Medicine, Vienna, Austria; 14 Department of Radiology and Nuclear Medicine, Rijnstate Hospital, Arnhem, The Netherlands; 15 Department of Biomedical Sciences, Humanitas University, Milan, Italy; 16 Department of Nuclear Medicine, Humanitas Clinical and Research Center, Milan, Italy; 17 Department of Radiology and Nuclear Medicine, Radboud UMC, Nijmegen, The Netherlands; 18 Department of Internal Medicine, Radboud UMC, Nijmegen, The Netherlands; 19 Endocrinology Service, Memorial Sloan Kettering Cancer Center, New York, NY USA; 20 Department of Nuclear Medicine, University Cancer Center Toulouse Oncopole, Toulouse, France; 21 Washington Hospital Center, Washington, DC USA

In 2017, the European Association of Nuclear Medicine, the Society of Nuclear Medicine and Molecular Imaging, the European Thyroid Association, and the American Thyroid Association established an intersocietal working group on differentiated thyroid cancer (DTC). The mission statement summarizes the purpose, prioritization of discussion topics, and goals of the working group.

The mission of the intersocietal working group is to provide a forum to discuss our differences in an open, honest, data-driven, respectful manner. Discussion should focus on areas of agreement and those disagreements which result in meaningful differences in clinical management. The group will propose potential solutions and strategies to address those differences.

The intersocietal working group continues to demonstrate a constructive spirit in which a rotating group of the world’s top-level specialists on the management of patients with DTC from the fields of nuclear medicine and endocrinology have committed to ongoing discussions, which will include meeting on a yearly basis. As set out in the “Martinique Principles” [1], this meeting, which was held for the second time in March 2019, seeks to identify differences of opinion, determines the basis for these differences, suggests ways to resolve disagreements and, when not resolvable, guides practicing physicians regarding the different approaches in the management of DTC and associated reasoning. The meeting also is meant to serve as a stimulus to intersociety communication, which will facilitate clarifying different viewpoints while also helping adopt uniform terminology. Furthermore, this meeting serves the important function of stimulating communication between the involved societies, seeking to build an understanding of mutual viewpoints. Thus, the group is intended to serve as a “think-tank” rather than a forum for the development of detailed guidelines. Specifically, the working group will not interfere with the formal guideline process of the respective societies. Nonetheless, the intersocietal working group intends to publish editorials or papers (as appropriate) describing their interactions and discussions with suggestions for further research to address important management issues as well as summaries of consensus and proposed recommendations reached.

During the 2019 meeting, in addition to discussions on specific management issues, there was an extensive discussion on how to proceed with future interdisciplinary collaboration. Therefore, a steering committee of representatives from each society was assigned to investigate the topics for future meetings. To secure fresh input and ideas, it was decided that delegates from each society will be chosen on the basis of the topics of discussion, achieving a rotation of delegates to provide continuity while avoiding redundancy. We also acknowledge that the medical care for patients with DTC is multidisciplinary, involving other specialties beyond the current groups represented. However, in the interest of fostering intensive and open exchange, we feel that the number of participants must be limited for the time being. In order to gain the perspective of relevant sister society expertise while maintaining a manageable and facile group, it was agreed that representatives from other societies would be invited as dictated by the needs for the topics to be discussed. Potential expansion of permanent sister society representation within the group will be reconsidered at a future time once the effort has matured and is on more solid footing. However, the working group is certainly open to receive suggestions for discussion topics from patients, clinicians, and other thyroid cancer-related organizations. In 2019, topical discussions included the following: (1) the criteria for success of I-131 therapy, (2) whether there are variations in peri-therapeutic diagnostics which may lead to differences in treatment, (3) the optimal tests to include for post-surgical diagnostic imaging to guide the decision on whether or not to pursue I-131 therapy and (4) the arguments in favour of and against empirical and dosimetry-based approaches for determining the activity of I-131 required to treat iodine-avid DTC metastatic disease.

Although there was wide agreement on these topics amongst the participants, as expected there were also differences of opinion. Despite extensive efforts to review the literature, the available evidence was found to be insufficient to provide any definitive guidance. Furthermore, while the meeting initiated very productive discussions of each of the topics, we could not arrive at consensus opinions on these important topics. Therefore, the unanimous decision was made to pursue more narrowly defined questions to allow for more in-depth discussion during future meetings of the intersocietal working group.

In accordance with the mission statement, the working group decided to consider several actions to further resolve points of discussion addressed during both the 2018 and 2019 meetings. These efforts are to be coordinated by designated members of the working group. Actions agreed upon include the following:

1. Designing a study to assess practice differences between centers regarding peri-operative risk-stratification. Parameters to be reviewed include specific tests and procedures performed before, during and after surgery which might impact the decision about whether or not to treat with radioiodine.
2. Providing a series of cases of DTC with increasing disease severity to appropriate experts in the working group and associated thyroid experts. Each clinician will be asked to recommend for or against I-131 therapy in each case and to recommend an appropriate therapeutic activity. This study should help better understand where disagreements in approach to therapy exist between the four societies members.
3. Providing a protocol for a trial on the benefit of dosimetry in metastatic, I-131-avid DTC will be considered. This likely would be a two arm randomized prospective trial comparing empiric fixed dosing to whole body/blood dosimetry guided activity selection. Preliminary calculations indicate that a sufficiently and relevantly powered two-arm, randomized trial comparing a dosimetry-based activity as high as safely administrable (AHASA) to a fixed activity appears to be feasible depending on the choice of primary endpoint.
4. Suggesting that the four societies cooperate to establish an international committee to address the evolving definition and management options for radioiodine refractory thyroid cancer.

The results of these efforts are expected to contribute to the group’s stated mission and to establish a solid foundation for continued fruitful collaboration. Ultimately, it is hoped that these deliberations will promote the societies developing a level of consensus that will result in the long-term benefit for our patients by achieving unified, optimized care.
